# Modelling relationships between juvenile brown trout (*Salmo trutta* L.) density and landscape characteristics: Integration of electrofishing data and spatial data to assess population status

**DOI:** 10.1111/jfb.70570

**Published:** 2026-07-24

**Authors:** Faye L. Jackson, Robert J. Fryer, Lawrence J. B. Eagle, Iain A. Malcolm

**Affiliations:** ^1^ Scottish Government Marine Directorate, Freshwater Fisheries Laboratory Pitlochry UK; ^2^ Scottish Government Marine Directorate, Marine Laboratory Aberdeen UK; ^3^ School of Geosciences, Kings College University of Aberdeen Aberdeen UK

**Keywords:** abundance, assessment, brown trout, capture probability, electrofishing, habitat

## Abstract

Brown trout (*Salmo trutta* L.) are ecologically and economically important, but like many salmonids their populations are declining. Trout exhibit a high level of phenotypic plasticity, ranging from freshwater residency to anadromy. This complexity causes problems for assessment and constrains application of adult catch‐based assessment methods that are commonly applied to other species such as Atlantic salmon. Juvenile methods provide one of the most promising approaches for the assessment of trout that can quantify the status of populations, identify pressures and guide evidence‐based management actions. This study collated and analysed ad‐hoc multi‐pass electrofishing data from across Scotland to model relationships between fish density and habitat, having accounted for the effects of variable capture probability. Given a lack of consistent data collection and recording, the habitat was characterised by landscape proxies derived from spatial data. The density model was used to derive expectations for healthy trout populations (i.e., a benchmark) that could be used for assessments across spatial scales. Capture probability exhibited a negative gradient between the south‐west and north‐east of Scotland, was generally higher for parr (>0+) than fry (0+), and higher on the first than subsequent passes. Capture probability increased over the summer for fry before levelling off in the autumn. The positive day of the year (DoY) effect was smaller and more linear for parr. Capture probability decreased with upstream catchment area (UCA), with the negative response being stronger for parr than fry. Altitude had a small negative (logistic)‐linear effect, which was common to both Lifestages. Trout densities (both fry and parr) were generally lower in the west and north of Scotland than in the east. Densities decreased non‐linearly with UCA, with stronger responses for fry than parr. Trout fry densities also declined strongly with altitude. However, trout parr densities exhibited a weak positive relationship with altitude and decreased with river distance to sea (RDS). There was no effect of RDS on fry densities. Fry and Parr densities both decreased with DoY. Two possible benchmarks were produced for each Lifestage. The first included all fixed effects. The second excluded regional effects on the assumption that the underlying abundance data contained spatial biases reflecting uncharacterised pressures. When combined with a suitable formal survey design, these benchmarks can support assessment of the status of juvenile trout populations in Scotland for the first time.

## 1 INTRODUCTION

Native brown trout (*Salmo trutta* L., hereafter trout) populations extend from Northern Norway to North Africa, exhibiting a wide range of life history strategies (Klemetsen et al., 2003) from river resident to anadromous (hereafter sea trout), with considerable diversity between. Anadromous parents can establish freshwater resident populations (Walker, 2006) and vice versa (Duval et al., 2021; MacCrimmon & Marshall, 1968) with the balance of different life history strategies thought to reflect complex interactions between genetics, environment and trade‐offs between migration costs and growth opportunities (Ferguson et al., 2019). In accessible rivers, sea trout often dominate juvenile production due to their greater size and fecundity (Jones et al., 2025; Rohtla et al., 2017), and juvenile densities can be higher as a result (Bohlin et al., 2001; Jones et al., 2019).

Trout are an important game fish, a valuable indicator of ecosystem health (Ayllón et al., 2012) and a central component of European Union Water Framework Directive (WFD) status assessments, especially in northern rivers characterised by low fish species diversity. Sea trout are often considered to be of particularly high ecological, economic and cultural value and are highly prized by anglers (Lui et al., 2019; Walker et al., 2006). Despite their importance, trout populations have received considerably less attention than Atlantic salmon (*Salmo salar* L., hereafter salmon) populations with which they co‐exist (Alexandre et al., 2025; Hagen et al., 2025) and are often an afterthought in terms of monitoring and management. Most data on trout population trends are obtained as a by‐product of sea trout fisheries (Adams et al., 2022; Fiske et al., 2024; Losee et al., 2024a; Mota et al., 2016), although some juvenile studies have been undertaken (Donadi et al., 2023; Tissot et al., 2025). Available information suggests that sea trout populations are in decline across much of the species' range (Adams et al., 2022; Dębowski, 2018; Jutila et al., 2006; Mota et al., 2016) and that juvenile abundances are also in decline in some locations, although with substantial regional variability (Donadi et al., 2023, Tissot et al., 2025). In Scotland, the number of sea trout caught by anglers has declined since the 1960s, with 2024 being the fourth lowest catch on record (Scottish Government, 2025). This is despite drastic reductions in net fisheries and associated commercial catches.

The sustainable management of trout populations requires quantitative assessments of population status (e.g., Alexandre et al., 2025; Jackson et al., 2025; Malcolm, Millidine, Glover, et al., 2019), the pressures affecting them (e.g., Fiske et al., 2024; Forseth et al., 2017; Jackson et al., 2025) and the likely efficacy of management actions (e.g., Louhi et al., 2016). The challenges around assessment are exacerbated by the spatial plasticity in trout life history strategies (Alexandre et al., 2025) that makes the application of catch (fisheries) based adult stock assessment approaches less appropriate than for other salmonid species such as Atlantic salmon (White et al., 2023).

In recognition of these challenges, the International Council for the Exploration of the Seas (ICES) established a sea trout Working Group (WGTRUTTA) to develop assessment models for sea trout populations that could be applied across the species range (ICES., 2024a). This group identified juvenile assessment methods as one of the most promising approaches for evaluating the distribution, abundance and status of sea trout populations (Alexandre et al., 2025). However, they also identified some technical challenges in such an approach. Firstly, suitable electrofishing and habitat data need to be collected to common standards and quality controlled (Alexandre et al., 2025, Malcolm, Millidine, Glover, et al., 2019, Millar et al., 2016). Next, it is necessary to quantify capture probability across organisations, habitats and time periods (requiring fully quantitative multi‐pass electrofishing) to provide accurate (unbiased and relatively precise) estimates of abundance (Dauphin et al., 2019; Glover et al., 2018; Millar et al., 2016). Finally, benchmark comparisons (i.e., the densities that should be expected if populations are in good status) need to be defined by modelling expected fish density as a function of habitat, excluding the effects of anthropogenic impacts where possible (Malcolm, Millidine, Glover, et al., 2019). By comparing observed juvenile abundance to benchmark expectations, it is possible to obtain a measure of population status (i.e., are the populations healthy or not). When combined with an appropriate survey design and modelling framework, population status can be reported at site, sub‐catchment, catchment, regional and national scales (e.g., Jackson et al., 2025; Malcolm et al., 2020; Malcolm et al., 2023; Malcolm, Millidine, Glover, et al., 2019; Malcolm, Millidine, Jackson, et al., 2019).

Previously published juvenile trout assessment methods have related abundance to geographic location and habitat quality (Pedersen et al., 2017), characterised by local field‐derived habitat characteristics (e.g., velocity, depth, substrate, cover). These methods have been applied to assess the status of sea trout populations across the Baltic (ICES, 2024b). However, such approaches rely on consistent field‐based habitat data collection, which is not always available in other European countries, including Scotland (Alexandre et al., 2025). Furthermore, these assessment benchmarks cannot be readily scaled across rivers in the absence of spatially continuous habitat data.

An alternative approach would be to model observed trout abundance in relation to landscape or macroscale characteristics (e.g., upstream catchment area, altitude, land use). These characteristics influence (or are proxies for) habitat availability and quality (Buffam et al., 2021; Foldvik et al., 2017; Frederico et al., 2014; Malcolm, Millidine, Glover, et al., 2019; Steel et al., 2016) or migratory costs, likelihood of anadromy and ova deposition (Bohlin et al., 2001; Jones et al., 2025). Where landscape characteristics can be calculated for all locations along a river network, it is possible to predict benchmark densities across varying spatial scales, as exemplified for salmon by Malcolm, Millidine, Glover, et al. (2019).

Electrofishing is the most common method for monitoring and understanding freshwater and diadromous fish populations in many countries (Alexandre et al., 2025). It is also the only sampling method capable of providing reliable catch‐independent and scalable estimates of abundance over varying spatial scales from individual sites to whole countries. In Scotland, electrofishing data are collected by various fisheries management organisations, often using varying site selection and field data collection procedures, making the data challenging to analyse and interpret.

This paper developed an assessment benchmark that can be used to determine the status of juvenile trout populations at a national scale, including sea trout populations in accessible rivers. The study made use of a large‐scale and long‐term (1997–2017) electrofishing dataset and statistical methods previously developed for assessing juvenile salmon in Scotland (Glover et al., 2018; Malcolm, Millidine, Glover, et al., 2019; Millar et al., 2016), where legacy data and ad‐hoc site selection pose particular analytical challenges. The specific objectives are the following:

1. Collate and characterise the availability of multi‐pass electrofishing data suitable for understanding trout populations.
2. Model spatio‐temporal variability in capture probability as a function of landscape covariates, location, time and covariates indicative of electrofishing teams and equipment.
3. Model spatio‐temporal variability in juvenile trout density as a function of landscape covariates, including the presence of migratory barriers, location and time, having accounted for capture probability.
4. Explore approaches for generating benchmark densities that could be used to assess the status of trout populations across varying spatial scales.

## 2 METHODS

### 2.1 Ethics Statement

The electrofishing data used in this study were initially collected for a wide range of management and assessment purposes by District Salmon Fisheries Boards, Fisheries Trusts, Government and Government Agencies. Fish capture methods were regulated under the Salmon and Freshwater Fisheries (Consolidation) (Scotland) Act 2003. Individual organisations complied with relevant legislation and licensing requirements for fish processing, depending on the purpose of sampling. In the case of sampling for assessment and management, no permits were required. Only data collected by the Scottish Government's Marine Directorate were covered under the Animals (Scientific Procedures) Act 1986 (ASPA). In all cases, fish were returned to the stream after sampling and enumeration.

### 2.2 Electrofishing data

Multi‐pass quantitative electrofishing data were collated from 25 fisheries management organisations across Scotland. Organisations collected data at various spatial and temporal scales for a range of different purposes. Local fisheries trusts and District Salmon Fisheries Boards typically collected data only within their area of responsibility (e.g., river catchment or hydrometric area), whereas organisations with a national remit (e.g., Marine Directorate, Scottish Government and Scottish Environment Protection Agency) collected data more widely but at fewer sites.

The electrofishing dataset used in the current study was broadly consistent with that used by Malcolm, Millidine, Glover, et al. (2019) to develop a juvenile salmon assessment benchmark. However, there were a few notable differences which resulted in more sites available for the current analysis. Specifically, sites located above migration barriers for salmon (*n* = 645, Figure 1) or where there was a chance of salmon stocking effects (*n* = 560) were retained in the current analysis. Furthermore, additional data were collated for 2017 to provide greater geographical coverage in that year. Consistent with previous analyses, site visits were excluded where there was evidence of partial sampling (e.g., edge of the river or a single channel of a braid).

**FIGURE 1:**
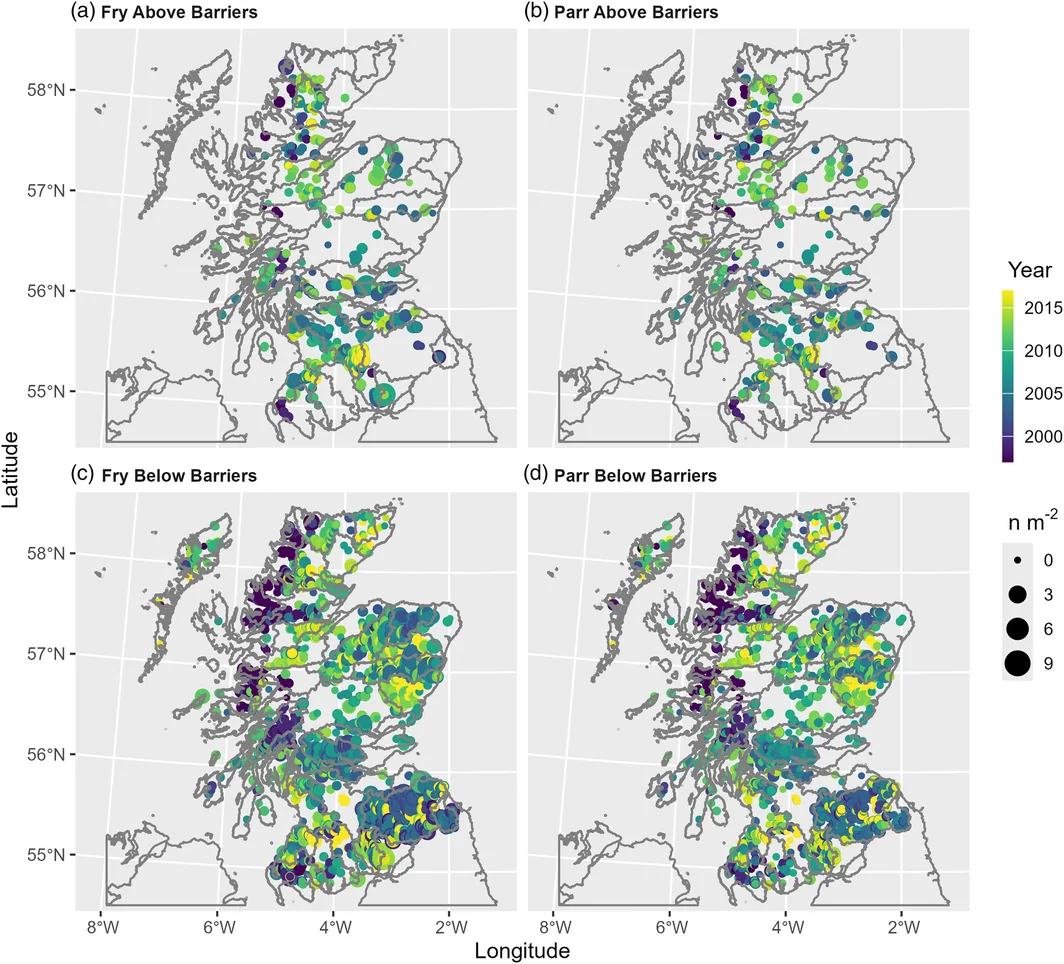
Data availability between 1997 and 2017. Columns show fry (0+) and parr (>0+) Lifestages, and rows indicate whether sites are located above or below barriers to salmon migration (in the absence of specific trout barriers datasets). Colours indicate the year of sampling. Point sizes indicate the estimated brown trout (*Salmo trutta* L) density obtained by application of the final capture probability model.

The final dataset included 6245 site visits to 2924 unique sites, between the years 1997 and 2017 (Figure 1). More recent data [e.g., data collected under the National Electrofishing Programme for Scotland (NEPS)] were omitted from the benchmark development to ensure that data which will be used for trout assessments (i.e., current observations) remain independent of the benchmark models. Furthermore, the use of the longer‐term legacy dataset also allowed for exploration of temporal trends that would not be possible with shorter time series. Only juvenile trout fry (0 year old) and parr (>0 year old) were included in the analysis. The few trout identified as adults by the data collection organisations were removed from the dataset prior to analysis.

### 2.3 Model covariates

Model covariates (Table 1) comprised spatial habitat predictors (e.g., landscape and river characteristics) and temporal covariates (e.g., Lifestage and Cohort) consistent with those described by Malcolm, Millidine, Glover, et al. (2019). These covariates have been shown to be useful predictors of capture probability and salmonid abundance in previous studies and to correlate with relevant reach‐scale habitat characteristics (Bryant & Woodsmith, 2009; Foldvik et al., 2017; Milner et al., 1998; Pépino et al., 2017; Steel et al., 2016; Thompson & Lee, 2000). Methods for calculating landscape covariates, underlying spatial datasets and relevant R packages are described in detail by Jackson et al. (2017). Additional covariates were included in the capture probability models to allow for differences in capture probability between the first pass and subsequent passes (Pass) and organisations, teams and equipment (Organisation).

**TABLE 1:** Model covariates, with description, unit and any transformation.

Covariate	Description	Units	Transfor‐mation	Type	Effect	Model
Alt	Altitude of the site	m	Square root	Cont	F	C + D
UCA	Upstream catchment area of the site	km^2^	Fourth root	Cont	F	C + D
RDS	River Distance to Sea	km	Square root	Cont	F	C + D
Gradient	Gradient of the site	%	Log	Cont	F	C + D
% Conifer	Percentage conifer in the riparian zone	%	/	Cont	F	C + D
% Mixed‐woodland	Percentage of mixed woodland in the riparian zone	%	/	Cont	F	C + D
Barrier	Site above or below a barrier to migration (Above, Below)	/	/	Cat	F	D
HA	Groupings of similar adjacent river catchments	/	/	Cat	F	C + D
Pass	Electrofishing pass fish were caught on (1, 2+)	/	/	Cat	F	C
Lifestage	Fish Lifestage (fry, parr)	/	/	Cat	F	C + D
Organisation	Grouping of organisations, teams and equipment	/	/	Cat	F	C
DoY	Day of the year from 1 January (1–365)	/	/	Cont	F	C + D
Year__Cont_	Year of sampling	/	/	Cont	F	C
Year__Cat_	Year of sampling	/	/	Cat	F	C
Cohort__Cont_	Year for fry and Year minus 1 for parr, linking the Lifestages of the same cohorts	/	/	Cont	F	D
Catchment	Rivers running to the sea	/	/	Cat	R	D
Site	Unique electrofishing site	/	/	Cat	R	D
Site‐visit	Unique visit to an electrofishing site	/	/	Cat	R	D
Lifestage	Fish Lifestage (fry, parr)	/	/	Cat	R	D
Cohort__Cat_	Year for fry and Year minus 1 for parr, linking the Lifestages of the same cohorts	/	/	Cat	R	D

*Note*: Transformations were applied to reduce skewness in the underlying covariate datasets. Type indicates whether the covariate is continuous (Cont) or categorical (Cat); Effect indicates whether it is a fixed (F) or random (R) effect; and Model indicates whether it is used in the capture probability model (C), the density model (D) or both (C+D).

All landscape covariates were calculated using R, version 3.2.3 (R Core Team, 2015). They were Altitude (Alt); Upstream Catchment Area (UCA); River Distance to Sea (RDS); Gradient, and Percentage Conifer (% Conifer) and Percentage Mixed woodland (% Mixed‐woodland) in the riparian zone. Altitude and RDS were square root transformed, UCA was fourth root transformed, and Gradient was log transformed to reduce skewness. Larger‐scale spatial structure was captured by the Hydrometric Area (HA) and Catchment. HA are groupings of similar adjacent river catchments, which give 47 similarly sized regions that cover Scotland. Catchment denotes rivers running to the sea. These are typically single rivers with a catchment area of >10 km^2^ or a river length of >1 km. Where rivers have a catchment area <10 km^2^ or length <1 km, these are grouped with adjacent rivers. Barrier was included as a two‐level categorical variable (Above, Below) to indicate whether electrofishing sites were above or below barriers to salmon migration (Buddendorf et al., 2019). Salmon barriers were used as there is no national dataset for trout barriers in Scotland.

Temporal covariates included the Year of sampling, Day of the Year (DoY) and Cohort (defined as Year for fry and Year minus 1 for parr). Year of sampling was included in the capture probability model first as a categorical fixed effect (Year__Cat_) and then as a continuous fixed effect (Year__Cont_) (see Section 2.3). Cohort was included in the density model as both a continuous fixed effect (Cohort__Cont_) to allow for temporal trends in density and a categorical random effect (Cohort__Cat_) to allow for variation in cohort strength within catchments (see Section 2.4). Lifestage was a categorical variable with two levels (fry 0+ or parr >0+ fish). Organisation and Pass were categorical variables used to account for differences in capture probability associated with staff and equipment, and electrofishing pass (first pass and subsequent passes), respectively.

### 2.4 Capture probability model

Capture probability was modelled using methods first reported in Millar et al. (2016). In brief, capture probability was modelled as a logistic function of covariates following a step‐up‐down model selection procedure starting from a ‘large’ model:

(1) $$\begin{array}{l} \text{logit}\;P\sim \text{Lifestage}+\text{Pass}+\text{Lifestage}:\text{Pass}+\text{Organisation}\\ \;+\;\text{Organisation}:\text{Pass}+{\text{Year}}_{\_\mathrm{Cat}}+\text{Organisation}:{\text{Year}}_{\_\mathrm{Cat}}\\ \;+s\left(\mathrm{Alt}\right)+\;s\left(\mathrm{UCA}\right)+s\left(\mathrm{RDS}\right)+s\text{(Gradient)}+s\left(\%\text{Conifer}\right)\\ \;+\;s\left(\%\text{Mixed}-\text{woodland}\right)+s\left(\mathrm{DoY}\right)+s\left(\mathrm{HA}\right) \end{array}$$

where P is the capture probability, ‘~’ denotes a function of, ‘:’ denotes an interaction and ‘s()’ denotes a smooth term. All the smooth terms were fitted with fixed degrees of freedom (as the available software cannot estimate the amount of smoothing from the data). The smooths [apart from the hydrometric area (HA) smooth] were constrained to two degrees of freedom, allowing at most a modal response. HA was modelled with a spatial smooth on 11 degrees of freedom, fitted using reduced rank Gaussian Markov random fields, to allow correlation between neighbouring regions (Millar et al., 2015, 2016). Changes in capture probability between years were first modelled using the categorical variable Year__Cat_ to allow for any pattern of change.

At each stage of the step‐up‐down model selection, it was possible to drop interaction terms involving categorical variables, replace smooth terms with linear terms and drop the main effects of categorical and continuous variables that were not involved in any interactions or expressed as smooth terms. Interactions could also be added, which allowed different relationships (and thus capture probabilities) between continuous variables and each Lifestage and/or Pass. Two‐way interactions were also considered between Lifestage and HA, and up to three‐way interactions between Lifestage, Pass and Organisation and between Lifestage, Year__Cat_ and Organisation.

Model selection was based on a modified form of the Bayesian Information Criterion (BIC_adj_) that incorporated an estimate of overdispersion (see Millar et al., 2016 for details). The overdispersion term accounts for ‘extra’ variation that would ordinarily be accounted for by random effects, but which is not possible in the available software. All models were fitted by maximum likelihood using the Marine Directorate in‐house R package ‘ef’ (Millar et al., 2017).

The categorical covariate Year__Cat_, used to identify any pattern of change in capture probability over years, was dropped in the model selection process. However, this term is heavily penalised because it has 20 degrees of freedom. Thus, the model space around the final model was explored further by introducing the continuous covariate Year__Cont_ as a linear term (and potentially a smooth term) and restarting the model selection process. Year__Cont_ is a more parsimonious covariate than Year__Cat_ and specifically investigates evidence of temporal trends in capture probability.

The effects of the covariates in the final model were illustrated by partial effects plots, where all covariates were held constant, except the individual covariate of interest. The importance of each model/term was calculated using the change in BIC_adj_ when the term was removed from the model, and significance was assessed using a chi‐squared test, adjusted by the overdispersion estimate.

### 2.5 Density model

Juvenile trout density modelling was consistent with the approaches outlined by Malcolm, Millidine, Glover, et al. (2019) for salmon. In brief, total counts of fry and parr in each site visit were modelled using a generalised additive mixed model with a Poisson distribution and a log‐link. The fixed effects were of the form

(2) $$\text{Count}\sim f\left(\text{Covariates}\right)+\text{Offset}$$

where ‘Count’ was the total number of fish of each Lifestage captured on each Site‐visit (summed across all passes) and f(.) was an additive function of the covariates. The Offset was an adjustment for the fished area and capture probability at each Site‐visit, which allowed covariate effects to be expressed as fish densities. The Offset was calculated as follows:

(3) $$\text{Offset}=\log\left(\text{Area}\right)+\log\left(P_{\mathrm{cum}}\right)$$

where P_cum_ was the cumulative capture probability taken from the final capture probability model (full details in Glover et al., 2018). Area was the area of the river sampled by electrofishing. Random effects were included to account for variation in fish density at nested spatial scales, which were Lifestage within Site‐visit, within Site and within Catchment. A Lifestage within Catchment: Cohort__Cat_ random effect allowed for temporal variation in cohort strength within catchments. The variance of the random effects could differ for each Lifestage.

Model selection followed a step‐up‐down procedure starting from a ‘large’ model with a fixed component.

(4) $$\begin{array}{l} \text{Count}\sim \text{Lifestage}+s\left(\mathrm{Alt}\right)+s\left(\mathrm{UCA}\right)+s\left(\mathrm{RDS}\right)+s\text{(Gradient)}\\ \;+\;s\left(\%\text{Conifer}\right)+s\left(\%\text{Mixed}-\text{woodland}\right)+s\left({\text{Cohort}}_{\_\text{Cont}}\right)\\ \;+s\left(\mathrm{DoY}\right)+\;s\left(\mathrm{HA}\right)+\text{Barrier}+\text{Offset} \end{array}$$

where s() denotes smooth terms with the degrees of freedom estimated from the data. HA was again modelled as a spatial smooth to allow correlation between neighbouring regions. Cohort__Cont_ was included as a continuous covariate to allow for a national temporal trend in density. The procedure for adding, simplifying and removing model terms was the same as for the capture probability model. The model scope included two‐way interactions between Lifestage and the continuous variables and between Lifestage and HA.

Model selection was based on a modified BIC, where the sample size was the number of site visits. All models were fitted by maximum likelihood using gamm4 version 0.25 (Wood & Scheipl, 2017). Partial effects plots were produced to illustrate the responses, where all covariates were held constant, except for the individual covariate of interest. Similarly, the spatial effect of each covariate (or combinations of covariates) was illustrated by predicting fish density across the Digital River Network (DRN), while holding other covariates at a constant value. The plots illustrate relative differences in abundance in relation to particular effects and should not be interpreted in terms of absolute values. The importance and significance of each term were assessed by determining the change in Bayesian information criterion (BIC) and likelihood ratio tests, comparing models with and without each term, respectively.

### 2.6 Benchmark densities

The benchmarks developed in this study were intended to be similar in concept to ‘intrinsic habitat potential’ used in salmonid studies elsewhere (e.g., Burnett et al., 2007), whereby predicted densities should reflect an average expectation in a particular river/habitat in the absence of major anthropogenic pressures, where the available habitat is fully utilised. Two possible benchmarks were produced for each Lifestage, which were then mapped to visualise predictions.

The first benchmark used all the density model fixed effects, including the spatial effect of HA, to predict across the whole river network. DoY was the only temporal term in the final model and was held constant at its median value (238–26 August in a non‐leap year). This benchmark (hereafter Mean Scaled Regional) assumes that the observed regional patterns of abundance captured by the HA effect are naturally occurring features of the data (e.g., due to spatial variability in geology, soils and hydrochemistry not captured by the other spatial covariates). Consequently, expected densities vary regionally and as a function of landscape covariates. The second benchmark (hereafter Mean Scaled) excluded the effect of HA from the predictions. This assumes that the underlying abundance data have been affected by spatial data biases; for example, some regions have always had less healthy trout populations than others over the relevant period (1997–2017). With this benchmark, expected densities vary only as a function of landscape covariates.

Because back‐transformed predictions from Poisson mixed models are negatively biased estimates of the mean response, it was necessary to scale benchmark predictions to observed densities (see Malcolm, Millidine, Glover, et al., 2019 for details). This scaling ensured that the overall site‐wise mean predicted density from the density model (i.e., across all site visits at all sites) was equal to the overall site‐wise mean ‘observed’ density from the electrofishing data.

## 3 RESULTS

### 3.1 Capture probability

The final capture probability model was as follows:

$$\begin{array}{l} \text{logit}\;P\sim \text{Lifestage}+\text{Pass}+s\left(\mathrm{DoY}\right)+\text{Altitude}+\mathrm{UCA}\\ \;+\;\text{Lifestage}:\;s\left(\mathrm{DoY}\right)+\text{Lifestage}:\;\mathrm{UCA}+s\left(\mathrm{HA}\right) \end{array}$$

Hydrometric Area was the most important model covariate (Table 2), which showed that, after accounting for landscape covariates, capture probability was typically higher in the south‐west than in the north of Scotland (Figure 2f). Lifestage and Pass were the second and third most important covariates (Table 2). Capture probability was generally higher for parr than fry (Figure 2a,c,d) and higher in the first electrofishing pass than in subsequent passes (Figure 2b). Capture probability of fry increased with DoY, levelling off in early September, with a smaller, more linear increase for parr (Figure 2c). The effect of UCA also differed between Lifestages, with a steeper reduction in capture probability for parr than fry as river size increased (Figure 2d). Altitude was the least important covariate in the model with a small negative (logistic) linear effect common to both Lifestages (Figure 2e, Table 2).

**TABLE 2:** Importance of covariates in the final capture probability model (from least to most important) determined by comparing the final model with simpler models where covariates (and their interactions) have been removed one at a time.

	D.F.	ΔBIC
Final model	21	
Altitude	20	12.1
Upstream catchment area (UCA)	19	56.6
Day of the year (DoY)	17	73.0
Pass	20	122.8
Lifestage	17	244.9
Hydrometric area (HA)	10	379.6

*Note*: D.F. indicates the model degrees of freedom after the covariate has been removed. All covariates were significant at *p* < 0.001. Higher ΔBIC values are associated with more important covariates.

Abbreviations: BIC, Bayesian information criterion; D.F., degrees of freedom.

**FIGURE 2:**
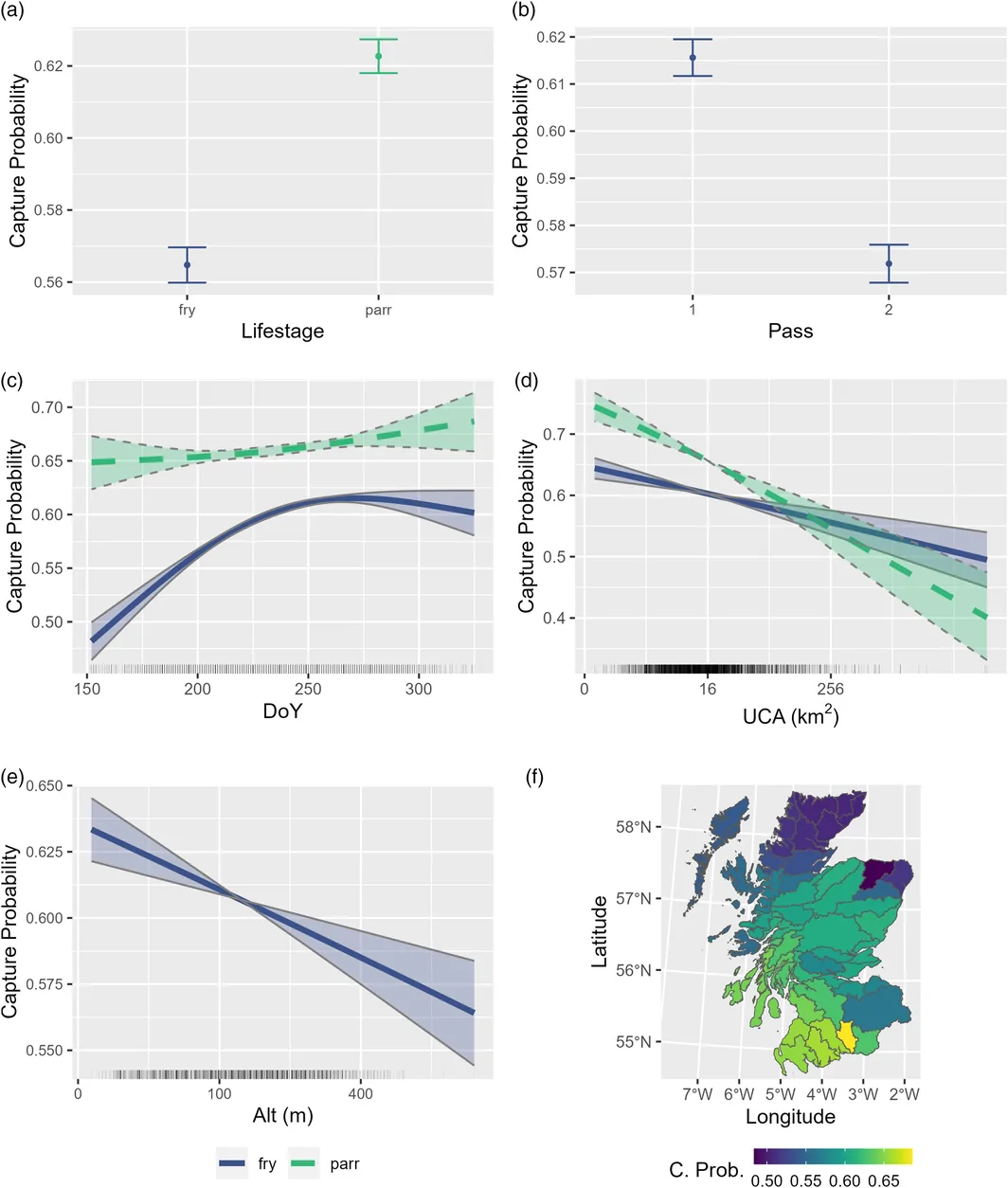
Partial effects from the final capture probability model. Effects of Lifestage (a), Pass (b), DoY (c), UCA (d), Alt (e) and HA (f) on capture probability. DoY and UCA effects differ between fry (solid blue) and parr (dashed green). All effects are scaled to the mean fitted first pass capture probability. Approximate 95% pointwise confidence intervals are shown as shaded areas or vertical lines. A rug indicates the distribution of the data on the *x*‐axis. Alt, Altitude; DoY, day of the year; HA, hydrometric area; RDS, River Distance to Sea; UCA, upstream catchment area.

### 3.2 Trout density

The final model of trout density was:

$$\begin{array}{l} \text{Count}\sim \text{Lifestage}+\mathrm{DoY}+\mathrm{RDS}+\mathrm{Alt}+s\left(\mathrm{UCA}\right)+\text{Lifestage}:\;\mathrm{RDS}\\ \;+\;\text{Lifestage}:\;\text{Altitude}+\text{Lifestage}:\;s\left(\mathrm{UCA}\right)+s\left(\mathrm{HA}\right)+\text{Offset} \end{array}$$

The effects of Alt, UCA and RDS differed between Lifestages, whereas the effects of DoY and HA were the same for fry and parr. UCA was the most important covariate with strong negative effects on abundance at higher UCA values for both Lifestages (Figure 3b, Table 3). Fry densities decreased monotonically with UCA, whereas parr densities increased initially (to ~5 km^2^) and then decreased monotonically (Figure 3b). Altitude had a similarly strong negative effect on fry density, but a small positive effect on parr density (Figure 3a). RDS had a negative (log‐)linear effect on parr density but had no effect on fry densities (Figure 3c). DoY had a small negative (log‐)linear effect of DoY on trout densities and was the least important of the model covariates (Figure 3d, Table 3). Having accounted for the effects of the landscape covariates, there remained a regional (HA) trend in density, which was the third most important covariate (Table 3). The HA effect produced a density gradient with lower densities in the north and west than in the east of Scotland (Figure 3e). The estimates of the variance components (Table 4) show additional variation in density, unaccounted for by the covariates, at all spatial scales, with the variation always greater for fry than parr.

**FIGURE 3:**
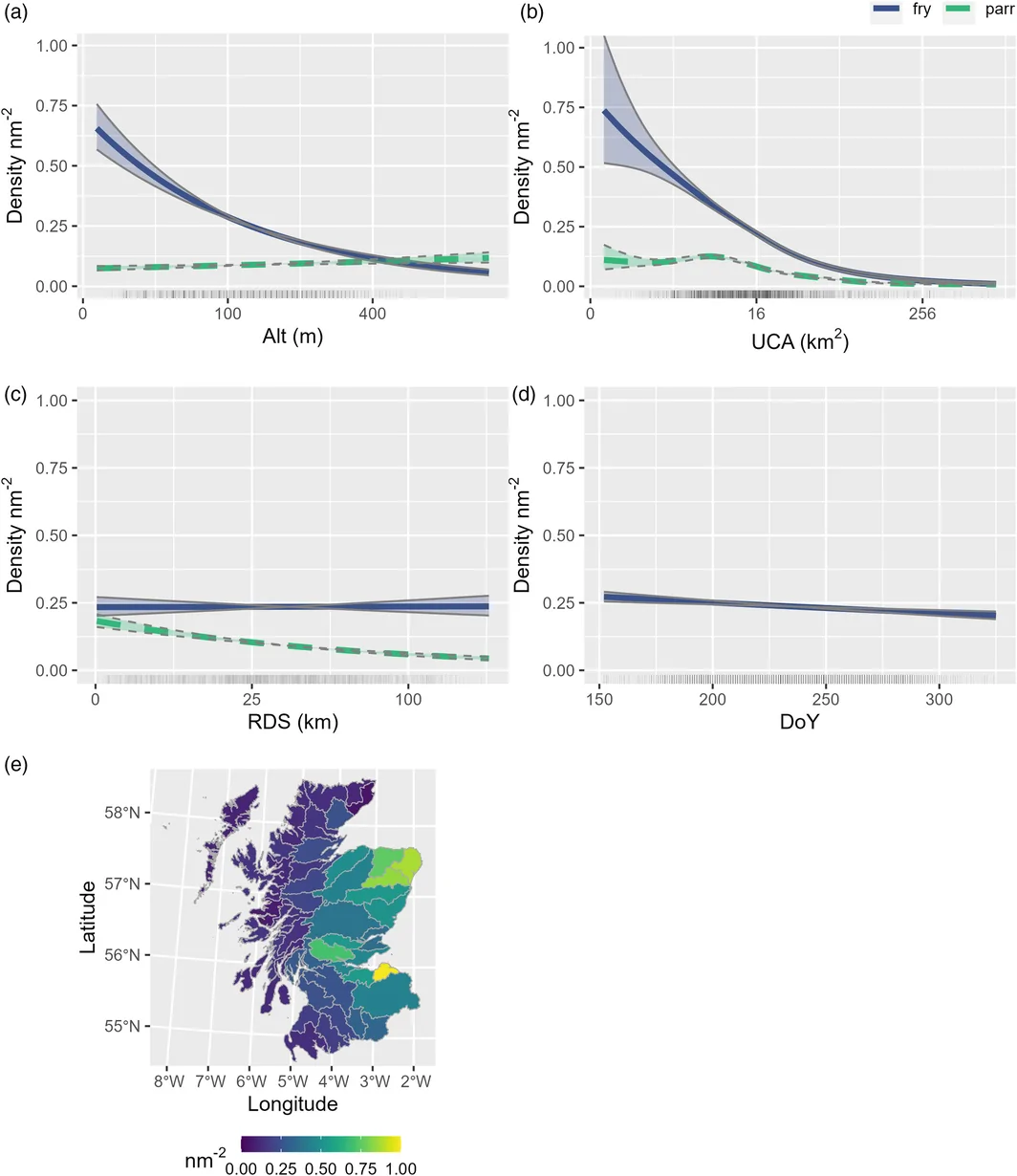
Density model partial effects. The effect of Alt (a), UCA (b), RDS (c), DoY (d) and HA (e) on brown trout (*Salmo trutta* L) density. The effects of DoY and HA are common across Lifestages and are scaled to the site‐wise mean observed fry density. RDS, Alt and UCA effects vary for fry (solid blue) and parr (dashed green) and are scaled to the site‐wise mean observed fry and parr density, respectively. Approximate 95% pointwise confidence intervals on the continuous effects are shown as shaded areas. A rug indicates the distribution of the data on the *x*‐axis. Alt, Altitude; DoY, day of the year; HA, hydrometric area; RDS, River Distance to Sea; UCA, upstream catchment area.

**TABLE 3:** Importance of covariates in the trout density model determined by comparing the final model with simpler models where covariates (including interaction terms) have been removed one at a time.

Model	D.F.	ΔBIC
Final	24	
DoY	23	4.9
RDS	22	29.2
HA	23	71.3
Altitude	22	88.0
Life stage	19	125.9
UCA	20	684.6

*Note*: The smoothers were fitted as variance components, so they were associated with a single degree of freedom. D.F. indicates the model degrees of freedom after the covariate has been removed. All covariates were significant at *p* < 0.001. Higher ΔBIC values are associated with more important covariates.

Abbreviations: BIC, Bayesian information criterion; D.F., degrees of freedom.

**TABLE 4:** Estimates of the variance components from the density model, giving the standard deviation (SD) for fry and parr and the correlation between them.

Variance component	SD fry	SD parr	Correlation
Site‐visit	0.79	0.59	0.13
Site	1.13	0.87	0.55
Catchment	0.77	0.38	0.69
Catchment: Cohort	0.49	0.37	0.55

The spatial effects of the landscape covariates on fry and parr density are shown at a national scale in Figures 4 and 5, respectively, with a finer scale illustration for the River Tweed in Figure 6. UCA had the greatest effect on trout densities and provides broadly consistent spatial patterns for fry and parr, with lower densities in larger rivers, which are found particularly in the east and in lower lying areas (Figures 4b, 5b and 6a,b). In the case of fry, this effect is partially countered by the negative effect of Alt (Figures 4a and 6c) since lower altitudes (higher densities) are often also associated with high UCA (lower densities) in valley bottom areas. For parr the patterns are reversed, with higher densities at higher altitudes, reinforcing some of the effects of low UCA around the mountainous edges of catchments (Figure 6d). RDS was only a significant effect for parr (Figures 5c and 6f). The spatial effect almost mirrored that of Alt, but was greater in size, such that the combined spatial effects of Alt and RDS (Figures 5e and 6h) looked similar to the effect for RDS alone. In addition to the effects of landscape covariates, there was also a regional (HA) spatial effect, which was consistent for both Lifestages. The HA effect reduced densities in western and northern rivers relative to the east coast rivers.

**FIGURE 4:**
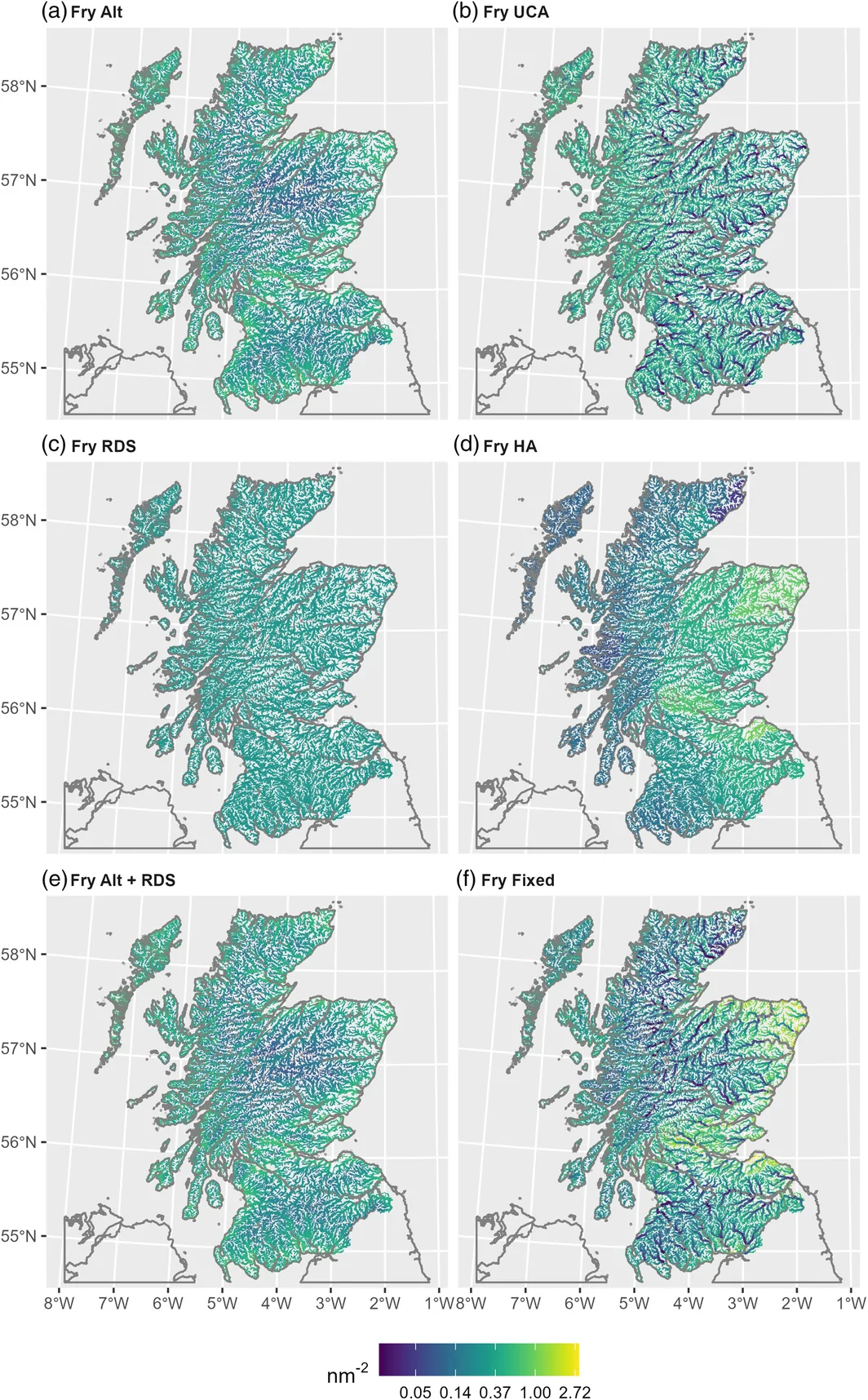
Spatial variability in the effects of Alt (a), UCA (b), RDS (c), HA (d), the combined effects of Alt and RDS (e) and all spatial fixed effects including HA (f) on brown trout (*Salmo trutta* L) fry density across Scotland. DoY was held at a median level (day 238). Predictions are scaled to the site‐wise mean observed fry density. Strahler order 1 rivers and lochs have been removed. Line thickness represents Strahler river order, with thicker lines being higher orders. Grey denotes where predictions have not been made (landscape covariates outside the environmental range of the electrofishing data). Alt, Altitude; DoY, day of the year; HA, hydrometric area; RDS, River Distance to Sea; UCA, upstream catchment area.

**FIGURE 5:**
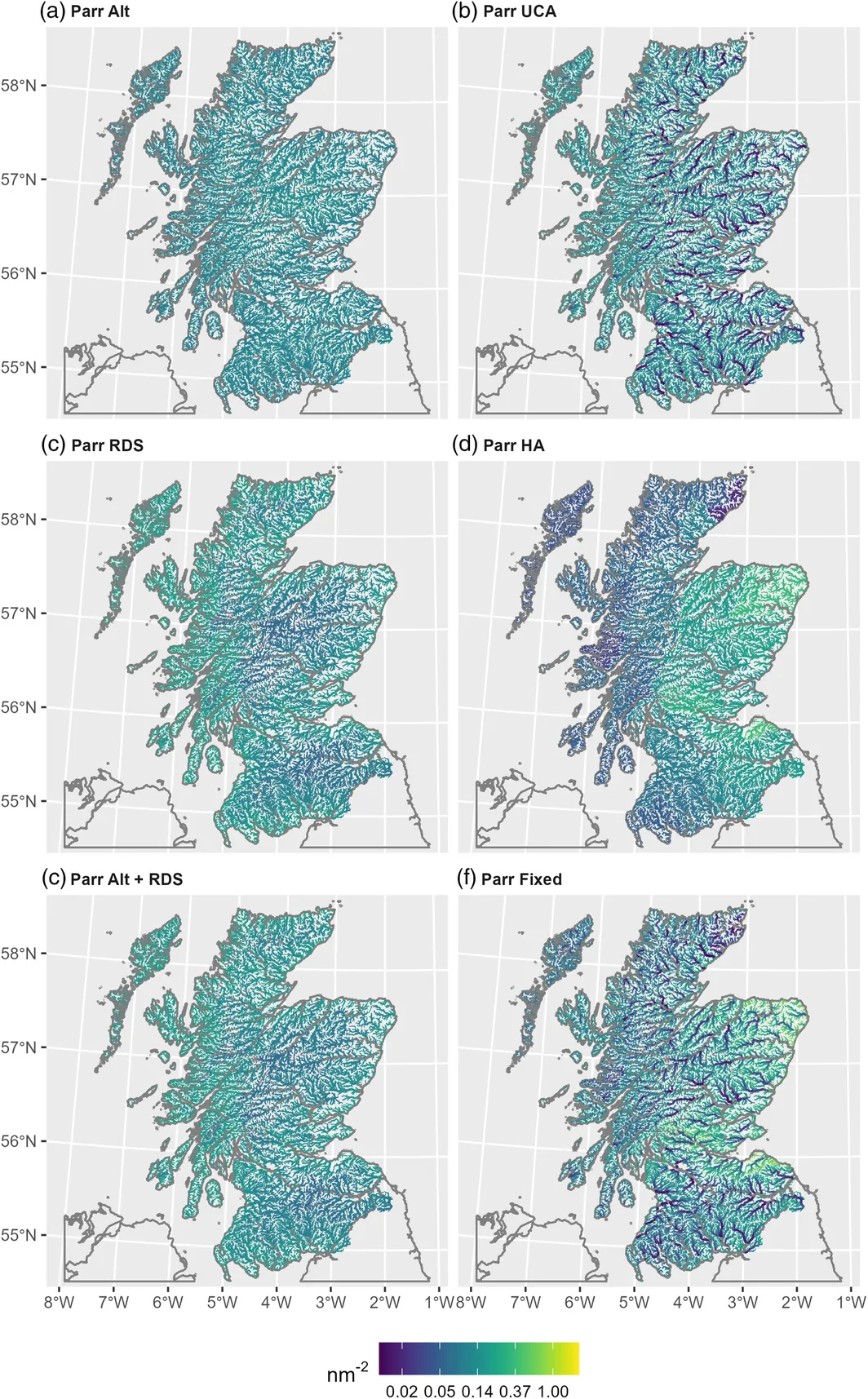
Spatial variability in the effect of Alt (a), UCA (b), RDS (c), HA (d), the combined effects of Alt and RDS (e) and all spatial fixed effects including HA (f) on brown trout (*Salmo trutta* L) parr density across Scotland. DoY was held at a median level (day 238). Predictions are scaled to the site‐wise mean observed parr density. Strahler order 1 rivers and lochs have been removed. Line thickness represents Strahler river order, with thicker lines being higher orders. Grey denotes where predictions have not been made (landscape covariates outside the environmental range of the electrofishing data). Alt, Altitude; DoY, day of the year; HA, hydrometric area; RDS, River Distance to Sea; UCA, upstream catchment area.

**FIGURE 6:**
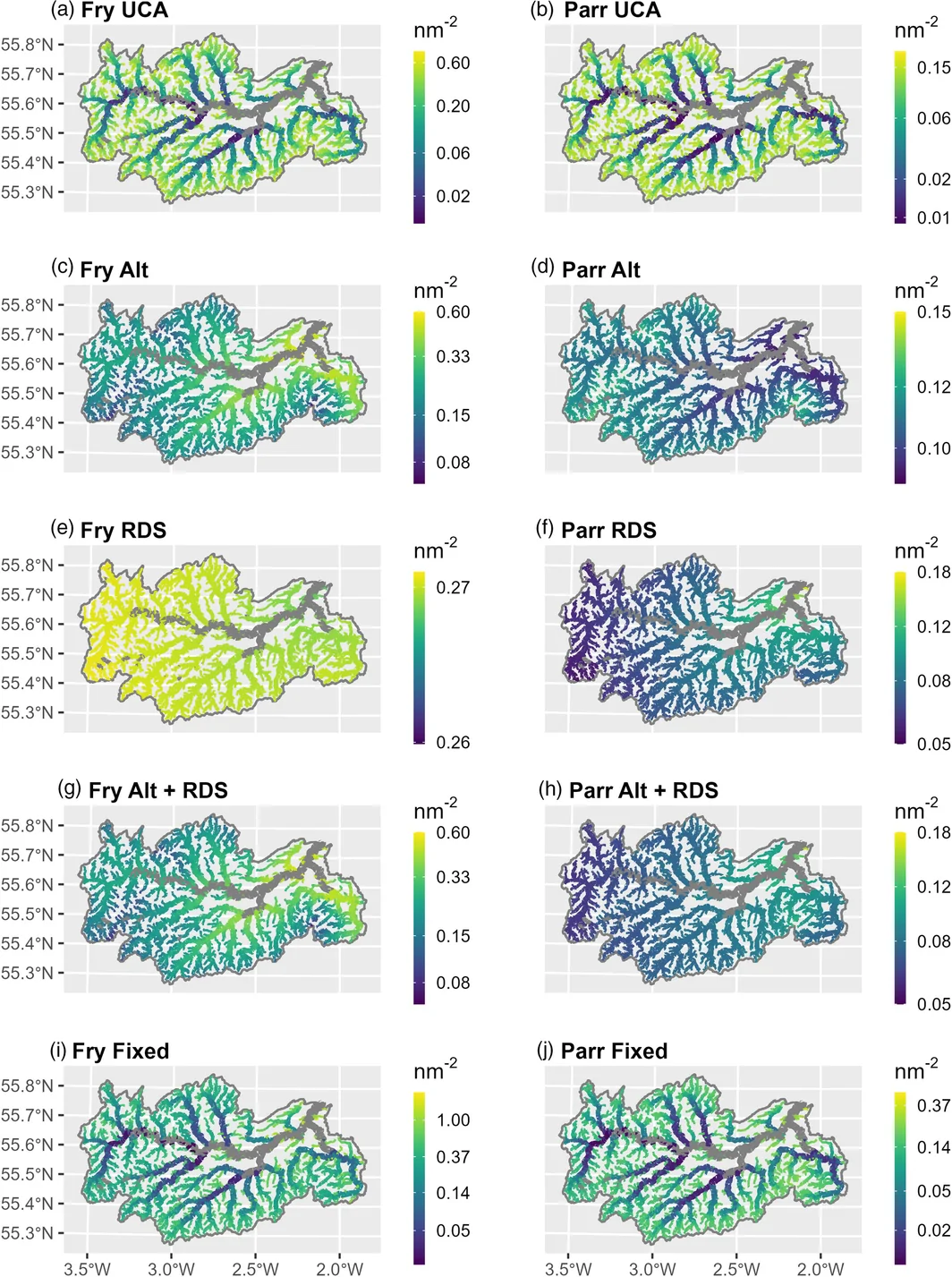
Spatial variability in the effect of UCA (a, b), Alt (c, d), RDS (e, f), the combined effects of Alt and RDS (g, h) and all spatial fixed effects (i, j) on brown trout (*Salmo trutta* L) density in the Tweed. The left column shows results for fry and the right for parr. DoY was held at a median level (day 238). Note that colour scales vary across plots. Predictions are scaled to the site‐wise mean observed density for the relevant Lifestage. Strahler order 1 rivers and lochs have been removed. Line thickness represents Strahler river order, with thicker lines being higher orders. Grey denotes where predictions have not been made (landscape covariates outside the environmental range of the electrofishing data). Alt, Altitude; DoY, day of the year; HA, hydrometric area; RDS, River Distance to Sea; UCA, upstream catchment area.

The overall spatial patterns of abundance produced by the combined fixed effects were similar for fry and parr (Figures 4f and 5f), with densities typically higher in the east (than north and west) and in smaller rivers close to the coast (Figure 6i,j). The main spatial differences between fry and parr density predictions were for high altitude locations near the coast, where parr densities were higher than those for fry.

### 3.3 Benchmark densities

Two potential assessment benchmarks were developed (Figure 7), where predictions of fry or parr densities were made from the density model, with and without regional hydrometric area effects (Mean Scaled Regional and Mean Scaled, respectively). The broad‐scale spatial patterns of abundance were similar between the benchmarks. Densities were highest in the east, with lower densities in the north and west. However, the Mean Scaled Regional benchmark showed a greater contrast, with higher densities predicted for lowland east coast rivers.

**FIGURE 7:**
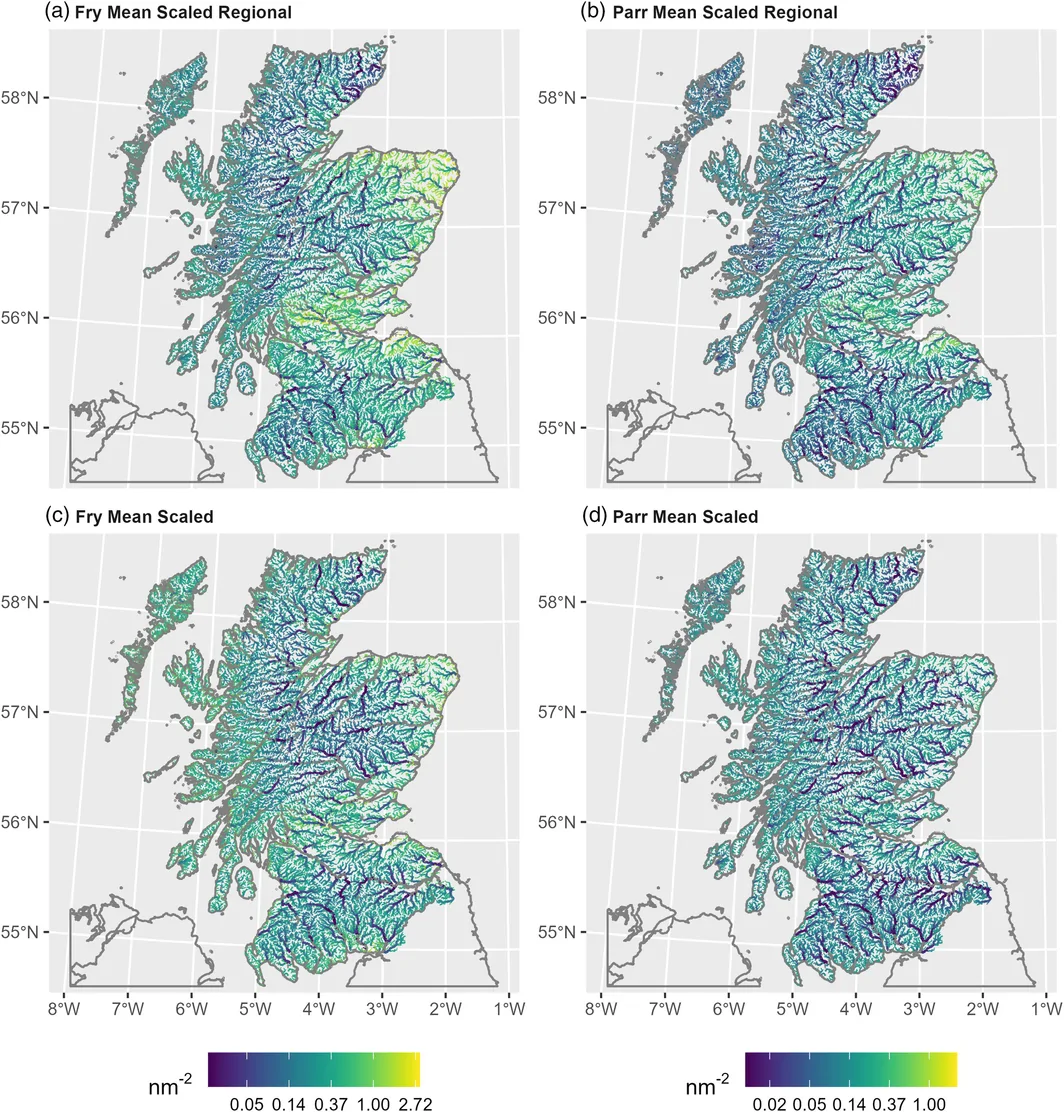
Development of benchmark densities for brown trout (*Salmo trutta* L) fry (left column) and parr (right column), showing where the HA effect is included (Mean Scaled Regional, panels a and b) or not (Mean Scaled, panels c and d). Low densities have been capped at 0.02 for fry and 0.01 for parr for plotting purposes. DoY was held at a median level (238). Grey denotes where predictions have not been made (landscape covariates outside the environmental range of the electrofishing data). Strahler order 1 rivers and lochs have been removed. Line thickness represents Strahler river order, with thicker lines being higher orders. DoY, day of the year; HA, hydrometric area.

When the regional effect is removed for the Mean Scaled benchmark (Figure 7c,d), the spatial variability is determined by the landscape covariates alone. Low altitude, smaller rivers (low UCA) that are close to the coast (short RDS) had the highest expected densities for both fry and parr.

## 4 DISCUSSION

Given the plasticity in life history traits exhibited by trout populations, juvenile assessment methods provide one of the most promising approaches for determining the status of populations, including anadromous components. However, there are several technical challenges in the development of such approaches, including a need to collate comparable electrofishing datasets, generate information on habitat characteristics, account for variability in capture efficiency, and quantify relationships between fish densities and habitat proxies. Juvenile assessment benchmarks have been successfully developed for salmon in Scotland (Malcolm, Millidine, Glover, et al., 2019) and for sea trout in accessible rivers in the Baltic (Pedersen et al., 2017). This study applied statistical methods and spatial data analyses to develop a large‐scale statistical habitat–abundance model for juvenile trout using a spatially and temporally extensive electrofishing dataset for Scotland. Density was modelled as a function of habitat proxies, having accounted for capture probability. The model was then used to develop two potential assessment benchmarks to determine the status of trout populations. These results are contextualised and discussed, alongside potential future developments below.

### 4.1 Capture probability

It is essential to account for differences in capture probability (over time, habitats, regions, people and equipment) to ensure unbiased and relatively precise estimates of fish density (Dauphin et al., 2019; Glover et al., 2019; Millar et al., 2016). Trout capture probability varied with Lifestage, Pass and DoY, with additional effects of HA, UCA and Altitude. Although several peer‐reviewed studies have documented similar effects for salmon (e.g., Malcolm, Millidine, Glover, et al., 2019; Malcolm, Millidine, Jackson, et al., 2019; Millar et al., 2016), few studies have considered the factors controlling capture probability in trout. Furthermore, there were some subtle differences in the trout capture probability model reported here compared to models of salmon capture probability that used a broadly similar dataset (Malcolm, Millidine, Glover, et al., 2019).

HA had the largest effect on capture probability. This could reflect spatial variability in habitat that was not characterised by the habitat proxies (Altitude and UCA), for example, large‐scale variability in water quality (Pregler et al., 2015), discharge and turbidity (Lyon et al., 2014), fish size (Hense et al., 2010) or density. However, it could also reflect the effect of Organisation, which was absent from the final model, and is partially confounded with HA (Millar et al., 2016) since Organisations and teams typically work within fixed geographic areas in Scotland. It is notable that Organisation was not in the final trout capture probability model, as previous studies have shown that different teams/staff (Benejam et al., 2012) and/or equipment (Dauphin et al., 2019) can have a substantial effect on capture probability. Furthermore, Organisation was the most important effect on capture probability in previously published Scotland‐wide studies of capture probability for salmon (Malcolm, Millidine, Glover, et al., 2019; Millar et al., 2016). It is therefore likely that the HA area effect in the current study reflects a mixture of organisation, equipment and habitat effects (not all of which have been characterised) that could not be disentangled.

The effects of Lifestage, Pass and DoY were the second, third and fourth most important effects on capture probability, respectively. These effects were consistent with previous studies and likely reflect the effect of varying fish size among other processes. Larger fish are more catchable (Benejam et al., 2012; Dolan & Miranda, 2003; Glover et al., 2020; Hedger et al., 2018; Hense et al., 2010); consequently, larger parr are caught more readily than smaller fry. Capture probability was also greater on the first pass than on subsequent passes (Benejam et al., 2012; Hedger et al., 2018; Millar et al., 2016; Peterson et al., 2004). This could reflect the removal of larger fish in earlier passes, although Dauphin et al. (2019) also showed that effort can reduce across successive passes as both fish abundance (fish available to catch) and staff attention are reduced. It is also possible that fish could be disturbed on early passes, relocating to areas where capture is more difficult (e.g., areas of greater cover). Capture probability increased with DoY, levelling off in early September for fry. This likely reflects fish growth over the summer months (Glover et al., 2019), especially for fry, where relative growth rates may be higher through early summer months (Dauphin et al., 2019; Letcher et al., 2023). However, it could also reflect changing environmental conditions, including increasing river temperatures (Millar et al., 2016; Pregler et al., 2015).

Capture probability also varied among habitats, as shown by the effects of UCA and Altitude. In common with previous studies, capture probability was lower in larger and wider rivers (higher UCA values) that are more challenging to sample efficiently (Dauphin et al., 2019; Hense et al., 2010). Altitude had a negative effect on capture probability. This potentially reflects a combination of discharge, temperature, and channel morphology effects on capture efficiency (Millar et al., 2016).

Previous studies have shown that capture probability can vary substantially between years (e.g., Malcolm, Millidine, Glover, et al., 2019, Malcolm, Millidine, Jackson, et al., 2019, Millar et al., 2016) and trend positively over time (Dauphin et al., 2019; Glover et al., 2018, 2019). In contrast, the final trout capture probability model reported here contained no Year effect. This could reflect differences in the underlying datasets, for example, fewer sites where repeat visits had sufficient trout to provide evidence of changes in capture probability. Alternatively, it could reflect differences in the precision of capture probability estimates (i.e., due to the number of fish caught) or greater stability in the sizes of juvenile trout relative to salmon, which have been shown to trend over time (e.g., Glover et al., 2019). Nevertheless, using multi‐pass data to check and account for potentially time‐varying capture probability remains prudent (Dauphin et al., 2019, Glover et al., 2019).

### 4.2 Trout density

Previous studies have shown that the habitat requirements of juvenile trout vary by age class and season (Ayllón et al., 2012; Millidine et al., 2012). Both Lifestages prefer coarser substrate (gravel, cobble, boulder) and in‐stream cover (e.g., algae, macrophytes, roots, undercut banks). Fry are generally found at shallower depths (0.2–0.4 m) and relatively high velocity areas. In contrast, parr are typically found at greater depths (0.4–0.7 m) and often use low velocity areas, especially in winter, although this depends on the availability of cover (Maki‐Petays et al., 1997). More generally, juvenile trout tend to thrive in environments associated with relatively cool water temperatures (Elliott & Elliott, 2010; Ojanguren et al., 2001), high dissolved oxygen, low acidity and high food availability.

At large spatial scales, the availability of suitable habitat is controlled by geology, glacial legacy, topography, climate and soils (Alexandre et al., 2025; Buffam et al., 2021; Donadi et al., 2023; Frissell et al., 1986). For example, latitudinal variability in climate (Caissie, 2006) and altitude (Jackson et al., 2018) can affect river temperature regime and thermal suitability for trout (Donadi et al., 2023). Similarly, geology and soils are primary controls on hydrochemistry (Smart et al., 2001), and the spatial distribution of physical habitat (morphological units, substrate, hydraulics) is strongly influenced by stream power and sediment supply, which in turn vary with channel gradient and catchment area (Addy et al., 2014; Montgomery & Buffington, 1997). Consequently, the spatial distribution of suitable habitat and thus juvenile densities might be expected to vary systematically across river systems (Gleason, 2015) with broad regional coherence, except where perturbed by local anthropogenic impacts (Fiske et al., 2024). These could include pressures such as land use change (Locke, 2024), channel modification and canalisation (Millidine et al., 2012), impoundments, weirs (Birnie‐Gauvin et al., 2017), point source pollution and alteration of hydrological systems through land drainage (Gramlich et al., 2018).

Large‐scale landscape characteristics can also influence the spatial distribution of anadromy by altering the fitness costs (energy, predation risk) and benefits (food availability, enhanced growth, fecundity) of migration. Where costs exceed the benefits associated with migration, selection processes are expected to favour residency over anadromy (Gross et al., 1988). Since anadromous females are generally larger than residents and associated with higher fecundity, spatial patterns of anadromy could therefore also affect abundance (Bohlin et al., 2001), particularly for fry where dispersal distances are typically in the order of a few 100 m (Palm et al., 2022).

Juvenile trout densities could therefore vary due to spatial differences in habitat quality (Armstrong et al., 2003), anadromy, and ova deposition, which are directly related (Bohlin et al., 2001), or both. Furthermore, the mechanisms controlling spatial variability in abundance could differ between Lifestages, depending on where population bottlenecks occur. For example, if juvenile production at the fry Lifestage was generally density independent, as seen by Glover et al. (2020) in the case of salmon, then large‐scale spatial variability in ova deposition could determine spatial variability in density, as habitats would rarely be saturated. In contrast, if physical habitat and density‐dependent processes constrained production of parr (through density dependence), then parr densities could largely reflect the suitability and quality of habitat.

In the current study, trout fry densities decreased with Altitude and UCA. In contrast parr densities increased with Altitude, showed a modal response to UCA, and declined with RDS. Both Lifestages increased from west to east (via the HA effect). The overall effect of these spatial variables was to increase densities in small (and medium‐sized in the case of parr) low altitude rivers, near the coast, particularly in the east and south‐east.

Several studies have observed negative relationships between trout fry density and altitude (Bohlin et al., 2001; Jones et al., 2019). This could be explained by spatial variability in productivity, gradient, and geomorphological characteristics, with high altitude locations being generally colder (Jackson et al., 2018), lower in nutrients (Jarvie et al., 2018), steeper, and with fewer ‘response’ reach types (Montgomery & Buffington, 1997) required by salmonids for spawning. However, it could also reflect lower ova deposition at altitude, with lower contributions from large, highly fecund anadromous or potamodromous trout. In a study of Swedish trout populations, Bohlin et al. (2001) showed that fry densities were greater and declined more sharply with altitude in anadromous than resident populations. Similarly, Jones et al. (2019) found that high fry densities were primarily found at low altitudes and hypothesised that this was due to anadromous contributions. Although trout fry densities did not show any significant relationships with RDS, this is often moderately correlated with altitude. In this context, Jones et al. (2025) studied the spatial distribution of trout fry with parents of anadromous origin and showed that densities decreased with distance from the sea.

Parr density had opposing relationships with Altitude (positive) and RDS (negative), with RDS having the greater effect size. The combined effect of these covariates was to increase densities at high altitudes close to the sea relative to rivers with similarly high altitudes further from the sea. This resulted in similar but subtly different spatial patterns of abundance for fry. In many cases, it is likely that high parr densities reflected the presence of multiple age classes. This could have arisen due to slow‐growing conditions at altitude or high migratory tendency in lower altitude rivers, close to the sea, consistent with low migratory costs, high competition and habitat saturation (Jones et al., 2019).

UCA had the greatest effect of the landscape covariates for both fry and parr. UCA is a proxy for river size that is in turn strongly correlated with hydraulic and sedimentary characteristics (Gleason, 2015), including higher widths, depths, and (to a lesser extent) velocities (Jackson et al., 2021; Leopold & Maddock, 1953) and fining of substrate. This results in habitat conditions in larger rivers that are generally less favourable to juvenile trout (Armstrong et al., 2003; Padoan et al., 2024; Peterson et al., 2004).

Having accounted for landscape effects, HA describes the remaining large‐scale spatial variability in juvenile trout density across Scotland. There was a west–east density gradient with lower densities in the north and west. In a previous analysis of juvenile salmon densities, the HA effect appeared to reflect anthropogenic pressures, with substantial density reductions around the urbanised ‘central belt’ in the south of Scotland, which could reflect historical and contemporary pressures (Malcolm, Millidine, Glover, et al., 2019). There is no apparent correlation with urban areas for trout, suggesting that the remaining spatial variability reflects either a large‐scale pressure that differs between the west and east of the country (Marine Scotland and Fisheries Management Scotland, 2023) or natural regional variability due to uncharacterised habitat controls.

Scotland is characterised by pronounced geological, topographical, hydrological and climatological gradients (Soulsby et al., 2002). There are marked west‐to‐east gradients in rainfall, runoff and evapotranspiration (Gilvear et al., 2002). Mean annual precipitation varies from 4000 mm in the highest altitude western areas to as low as 600 mm in lowland areas on the east coast (Soulsby et al., 2002), with consequences for the hydrological regime. The west coast is also associated with many smaller, steeper river catchments. Contrasts in geological and climatological characteristics also have a substantial effect on predominant soil types. Extensive blanket peat and peaty gleyed podzols dominate the west, south‐west and north, whereas semi‐podzols, podzols and related gley soils dominate in the north‐east (see Figure 2, Soulsby et al., 2002), which is where the HA effect is largest. Such patterns can strongly influence spatial variability in water quality (Jackson et al., 2025) with potential consequences for trout survival and abundance. For example, Almodóvar et al. (2006) found greater trout production in fertile rivers with high inorganic nutrient levels and in hard‐water streams. Similarly, Malcolm et al. (2014) found that trout were more likely to be absent from areas of high acidity, and Buffam et al. (2021) suggested that high organic acidity (due to high dissolved organic carbon) excluded trout from headwater streams. There is therefore a strong possibility that the effects of HA reflect the effects of natural environmental gradients that are important to trout abundance but not characterised in the current analysis.

Finally, the presence of physical barriers to migration (identified as impassable for salmon) did not appear to be a strong effect on juvenile trout abundance and was not included in the final model. This contrasts with previous studies where juvenile densities of trout above barriers were lower (Bohlin et al., 2001). There are several potential reasons for this. First, some of the sites above barriers in our analysis were in the upper parts of the river catchment (high Altitude and RDS), where lower densities are to be expected anyway. Second, barriers to salmon might be passable to trout. Finally, habitat suitability rather than ova deposition might constrain juvenile trout abundance across some Scottish rivers. Alternatively, the presence of nearby standing waters (not included in this analysis) could result in high ova deposition independent of access to sea trout.

### 4.3 Developing benchmarks for trout density

Regardless of the precise mechanisms controlling the spatial distribution of juvenile abundance, assessment benchmarks are required to identify pressures and manage populations. In the current analysis, we used landscape covariates to predict large‐scale variability in expected trout abundance. The final model did not include a trend for Cohort year, suggesting that, over the period 1997–2017, juvenile trout abundance was relatively stable, and declines in the catch (as a proxy of abundance) of sea trout did not affect juvenile density. Burnett et al. (2007) argued that abundance models based on landscape covariates can describe ‘intrinsic habitat potential’, while Firman et al. (2011) argued that the use of coarser‐scale, relatively static landscape features is more suited to the prediction of future habitat potential, as it ignores the current condition of local habitat. Accepting these arguments, challenges remain in converting abundance models into a reliable assessment benchmark, including whether or not to incorporate individual fixed effects in the benchmark predictions.

The approach presented here follows that of Malcolm, Millidine, Glover, et al. (2019), who constructed benchmark densities of juvenile salmon in Scotland. Two candidate benchmarks are derived with (Mean Scaled Regional) and without (Mean Scaled) the regional hydrometric area effect. In the case of salmon, the HA effect appeared to reflect historical and contemporary anthropogenic pressures around the urbanised ‘central belt’ and south‐west of Scotland, and the benchmark excluding HA was subsequently adopted for management (Malcolm, Millidine, Jackson, et al., 2019). In the current trout analysis, there was broad consistency in the large‐scale spatial density patterns between the two candidate benchmarks, with west–east gradients being accentuated when including HA. There are major topographic, climate, geological and water quality gradients across Scotland that could explain these gradients. Consequently, it is considered appropriate to use the benchmark, including HA, for trout assessments. Should future work identify a strong association between the HA effect and pressures not incorporated in the current analysis, then the choice of benchmark should be revisited.

This study was focused on the development of a habitat—abundance model suitable for establishing a scalable juvenile trout assessment benchmark. Illustrating how the model can be applied across spatial scales is beyond the scope of the current study. However, given appropriate spatial data, the benchmark can be readily scaled to catchments or regions by predicting densities for river segments and averaging these regionally, weighting river segments by their length (mean regional density). Alternatively, it is possible to sum the product of river width, length and density for each segment to obtain regional estimates of juvenile benchmark production. The status of populations can then be assessed by comparing observed and expected densities or production. Where reliable (unbiased and relatively precise) assessments of contemporary abundance are required, this is best achieved through an appropriate statistical survey design, such as the NEPS (Malcolm, Millidine, Jackson, et al., 2019), which is supported by a Generalised Random Tesselation Stratified (GRTS) survey (Stevens & Olsen, 2004).

### 4.4 Wider applicability and prospects for international juvenile trout assessment

While the modelling approaches and benchmarks developed here and in previous studies (Malcolm, Millidine, Glover, et al., 2019) were designed to address Scotland's particular fish assessment needs and data landscape, the approaches are more generally applicable. For example, the use of landscape covariates to predict capture probability and abundance in Scotland was necessitated by a lack of consistently recorded local habitat data, ad‐hoc data collection and a need to scale benchmarks across river networks for assessment purposes. However, similar challenges have faced ICES WGTRUTTA in their attempts to develop assessment methods for sea trout at European scales (Alexandre et al., 2025). In that context, the current study offers a potential template for future work.

Over the last 3 years, WGTRUTTA has been working to develop and compile datasets comparable to those employed in this study. This has included the development of a European dataset on barriers to define accessible rivers for anadromous trout and the collation of multi‐pass electrofishing datasets from across Europe. When these resources are combined with a new open‐access, topologically correct DRN with habitat covariates (French & McGinnity, 2026) and the types of modelling tools and approaches presented in this study, this offers a promising approach for defining benchmark expectations for sea trout at a European level. This work is at an early stage and will require careful development to explore whether the effects of landscape covariates are consistent and transferable or vary regionally. Nevertheless, the parts of a modelling framework are now in place, offering development opportunities for large scale application that did not previously exist.

### 4.5 Limitations and future work

The current study focused on the development of a benchmark which can be readily scaled across river networks using spatial data and can be derived from a wide range of electrofishing data sources in the absence of consistent local habitat data collection (Alexandre et al., 2025). Such an assessment benchmark is powerful for assessing the status of fish populations (on average) across catchments and regions with numerous electrofishing observations. However, such benchmarks are less useful for assessing the status of individual ad‐hoc electrofishing sites where local habitat variables can cause considerable fine‐scale variation in abundance. Water chemistry (Malcolm et al., 2014), substrate and flow/habitat types can all cause substantial local variability in salmonid abundance (Armstrong et al., 2003). In the future, it may be possible to produce a second suite of statistical models to improve predictions of densities for individual sites, provided that habitat data collection procedures can be harmonised at the time of sampling (Alexandre et al., 2025, Malcolm, Millidine, Glover, et al., 2019). Such models may also improve management of stressors by identifying pressure‐response relationships and defining appropriate reference conditions.

Developments in remote sensing technologies may also create opportunities for improved riverine habitat characterisation across rivers, allowing continuous classification of habitats across riverscapes (Woodget et al., 2017). Furthermore, there is potential to integrate large‐scale habitat and fish density models, where they can both be applied across similar spatial scales. For example, thermal regime is known to be an important control on trout densities, and Jackson et al. (2018) have produced a national‐scale maximum daily river temperature model. Predictions from this temperature model could be included as covariates in future salmonid density models.

The phenotypic plasticity of trout has been a persistent challenge for scientists aiming to produce reliable trout assessment methods. This study outlined a pragmatic approach for the assessment of trout populations, including freshwater resident, potadomorous and anadromous components, recognising that in accessible rivers the observed fish will comprise all components to varying degrees. Nevertheless, quantifying these contributions remains a central challenge for future work (Charles et al., 2004; Walker et al., 2006) and could substantially improve our understanding and prediction of juvenile trout densities. Charles et al. (2004) illustrated how carbon and nitrogen stable isotope analysis of ‘known origin’ tissue samples and emerging fry could provide insights into anadromy, but also highlighted the potential confounding influence of local physiochemical processes in interpreting outcomes. Genetic methods also present opportunities for improving our understanding of life history, either through adaptive markers (Lemopoulos et al., 2018) or genetic identification of progeny from adults of known origin (Duval et al., 2021). Assuming the propensity for anadromy could be reliably linked to genetic markers, and that these result in migration in a consistent way (e.g., with a known and reliable environmental factor, Reed et al., 2010), then the likelihood of anadromy could potentially be modelled from a combination of genetic and environmental variables to predict relative contributions of different trout life histories.

Although different salmonid species exhibit habitat preferences, their habitat requirements and distributions overlap (Armstrong et al., 2003). In particular, interactions and competition are to be expected between salmon and trout across freshwater environments (Armstrong et al., 2003). The current assessment benchmark for salmon in Scotland (Malcolm, Millidine, Glover, et al., 2019) and the benchmark developed in this study do not consider the potential for interspecific interactions to affect distribution and abundance. This largely reflects the technical challenges in fitting models to multiple species and Lifestages, with the need to incorporate additional interactions between species and covariates and error terms with more complicated correlation structures. Nevertheless, the potential consequences of interactions between trout and salmon (or other competitive species) for predicting abundance and benchmark expectations could be explored further using simpler models, for example, where the density of competitive species was included as a covariate. Such information may also be useful in the context of management or conservation action which targets a particular species or habitats, for example, through the addition of large wood.

Finally, the benchmark developed here assumes that future trout habitat requirements, spatial distribution and abundance will be consistent with historical norms (1997–2017). This might not be the case under rapid and irreversible climate change. Future assessment methods, conservation and management actions will need to understand the potential impacts of changing environmental (Free et al., 2024) and population processes (Crozier & Siegal, 2024; Free et al., 2024; Reed et al., 2010).

## 5 CONCLUSIONS

Assessment methods are required to understand and manage salmonid populations. However, in the case of trout, phenotypic plasticity poses particular challenges that have so far constrained development. Juvenile methods based on habitat—abundance models that assess expected densities irrespective of life‐history origin offer a pragmatic approach, but are associated with data and analytical constraints associated with historical electrofishing datasets. This study developed a national scale juvenile density model for Scotland having accounted for spatio‐temporal variability in capture probability. Densities of fry and parr were predicted from landscape variables indicative of habitat quality and environmental likelihood of anadromy. From this model, it was possible to produce benchmark expectations that can be applied at spatial scales ranging from individual sites to the whole country. When combined with an appropriate spatial survey of contemporary trout densities and information on pressures, this assessment tool can support targeted evidence‐based management of rivers and fisheries. Future work should seek to harmonise data collection protocols, including the collection of data on physical habitat, water quality and anthropogenic pressures. This could improve assessments of fish populations at individual sites and provide a process‐based understanding of the underlying drivers of regional variability currently represented by HA effects.

## AUTHOR CONTRIBUTIONS

**Faye L. Jackson:** Data Curation; formal analysis; software; validation; visualisation; writing—original draft preparation; writing—review and editing. **Robert J. Fryer:** Formal analysis; methodology; software; validation; writing—review and editing. **Lawrence J. B. Eagle:** Writing—review and editing. **Iain A. Malcolm:** Conceptualisation; data curation; formal analysis; methodology; software; resources; supervision; validation; visualisation; writing—review and editing; project administration; funding acquisition.

## FUNDING INFORMATION

This work was funded by the Scottish Government, Marine Directorate under Service Level Agreement FW02g and ADP739.

## CONFLICT OF INTEREST STATEMENT

The authors have no conflicts of interest to declare.

## Data Availability

The data underpinning this study have commercial value to the various charitable organisations that have contributed to the development of this study. The data were provided specifically for the purpose of developing assessment methods and tools, and were assured that raw data would not be shared publicly. Consequently, requests for data should be made directly to the data providers via the Scottish Fisheries Coordination Centre general@fms.scot.
